# Publisher Correction: CD146-HIF-1α hypoxic reprogramming drives vascular remodeling and pulmonary arterial hypertension

**DOI:** 10.1038/s41467-019-12107-7

**Published:** 2019-09-05

**Authors:** Yongting Luo, Xiao Teng, Lingling Zhang, Jianan Chen, Zheng Liu, Xuehui Chen, Shuai Zhao, Sai Yang, Jing Feng, Xiyun Yan

**Affiliations:** 1 Beijing Advanced Innovation Center for Food Nutrition and Human Health, China Agricultural University, Yuanmingyuan West Road 2, 100193 Beijing, China; 2 State Key Laboratory of Cardiovascular Diseases, Fuwai Hospital, National Center for Cardiovascular Diseases, Chinese Academy of Medical Sciences and Peking Union Medical College, No. 167 North Lishi Road, 100037 Beijing, China; 3 Department of Rheumatology and Clinical Immunology, Peking Union Medical College Hospital, Chinese Academy of Medical Sciences & Peking Union Medical College, No. 1 Shuaifuyuan, 100730 Beijing, China; 4 Key Laboratory of Protein and Peptide Pharmaceutical, Institute of Biophysics, Chinese Academy of Sciences, 15 Datun Road, 100101 Beijing, China; 5 Laboratory Animal Research Center, Institute of Biophysics, Chinese Academy of Sciences, 15 Datun Road, 100101 Beijing, China

**Keywords:** Hypertension, Respiratory tract diseases

Correction to: *Nature Communications* 10.1038/s41467-019-11500-6, published online 7 August 2019.

The original HTML version of this Article contained errors in Figures 6 and 7. Figures 6 and 7 were inadvertently replaced by incorrect figures. This has been corrected in the HTML version of the Article.

