# Supplementary material for: CD146-HIF-1α hypoxic reprogramming drives vascular remodeling and pulmonary arterial hypertension
Source: Nat Commun. 2019 Aug 7;10:3551. doi: 10.1038/s41467-019-11500-6 (PMC6686016; doi:10.1038/s41467-019-11500-6)
Supplement: Supplementary file 1 — Supplementary Information [file 41467_2019_11500_MOESM1_ESM.pdf]

Supplementary Information

CD146-HIF-1α hypoxic reprogramming drives vascular remodeling and pulmonary arterial hypertension

Luo et al.

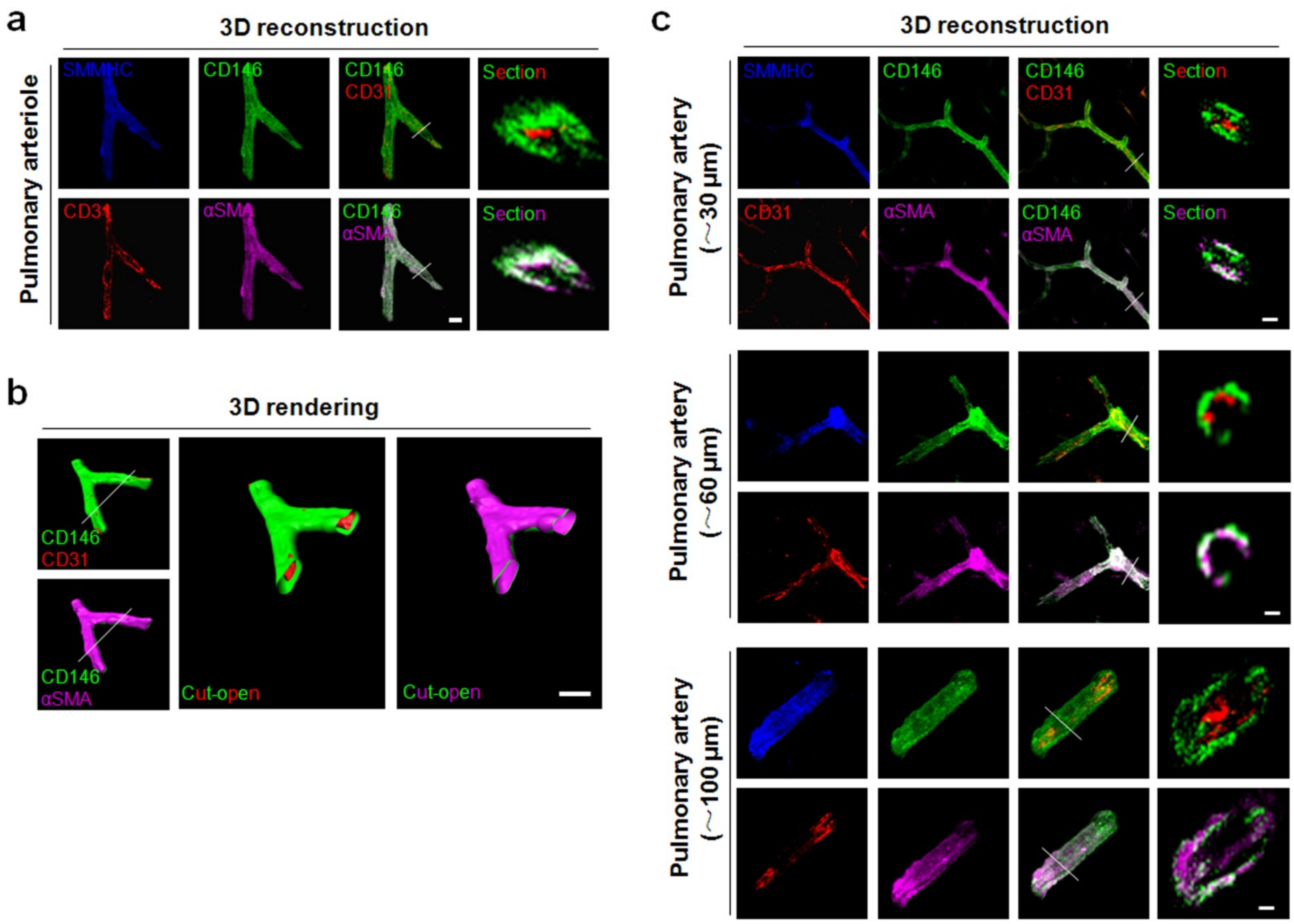

Supplementary Figure 1 Expression of CD146 in pulmonary arteries. **a**, **b** Lung sections (20 μm thickness) were stained for CD146 (green), CD31 (EC marker, red), arterial markers SMMHC (blue) and αSMA (purple). Shown are three-dimensional reconstructions of confocal image z-stacks of pulmonary arteries (**a**) and the three-dimensional surface rendering of epifluorescence images (**b**). White lines indicate cutting sites shown on the right of each panel, confirming that the expression of CD146 was exclusively observed in smooth muscle cells and not in PAECs of pulmonary arteries. Cut-open images were then created from the three-dimensional surface rendering of vessels to reveal the inner vessel wall (**b**). **c** Three-dimensional reconstructions of confocal image z-stacks of pulmonary arteries with differential vessel diameters. Scale bars represent 30 μm. At least 20 pulmonary arteries were analyzed.

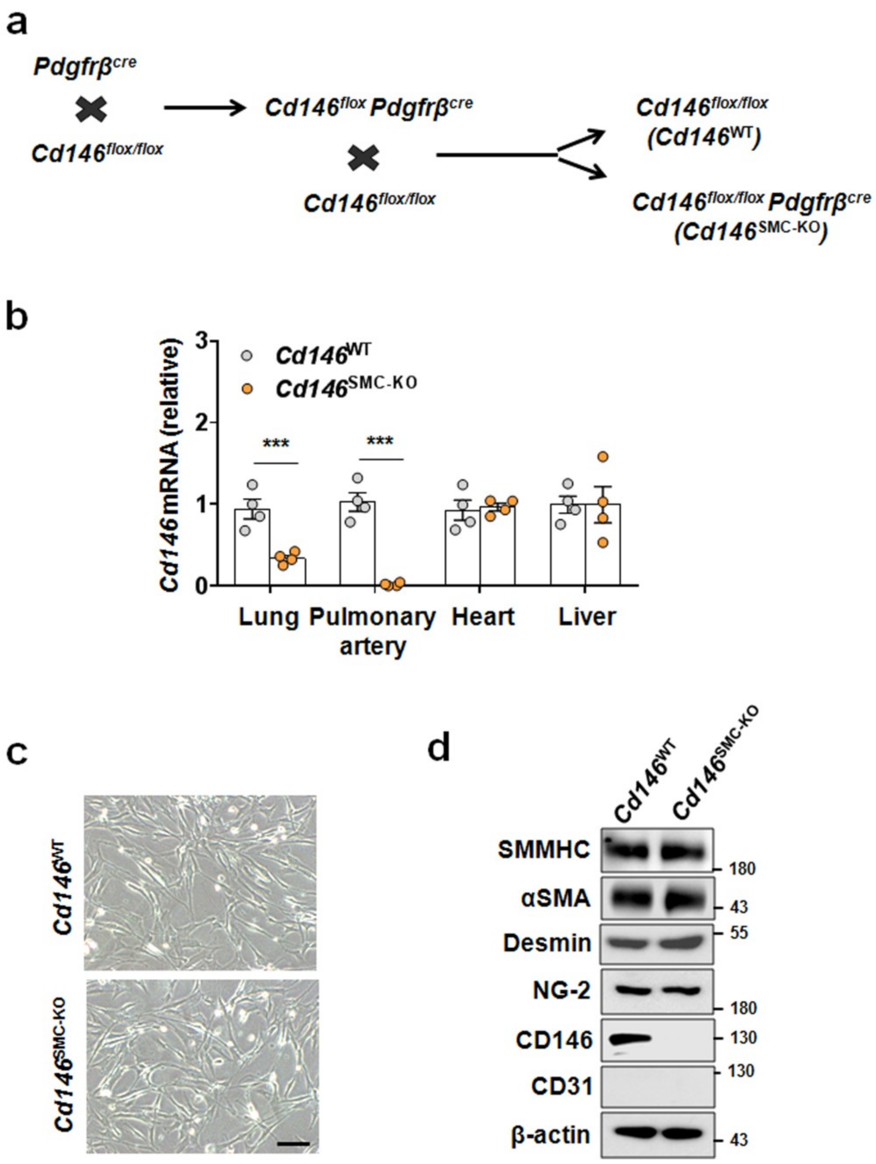

Supplementary Figure 2 Generation of smooth muscle cell-specific *Cd146* knockout mice. **a** Mating scheme to generate mural cell-specific *CD146* knock-out mice ($Cd146^{flox/flox}Pdgfr\beta^{cre}$, namely $Cd146^{\text{SMC-KO}}$) and control WT littermates ($Cd146^{\text{flox/flox}}$, namely $Cd146^{\text{WT}}$). **b** *Cd146* mRNA expression in lung, pulmonary artery, heart and liver from $Cd146^{\text{SMC-KO}}$ and $Cd146^{\text{WT}}$ mice (n = 4 biologically independent tissue samples per group). **c** Morphology of primary murine PASMCs isolated from $Cd146^{\text{WT}}$ and $Cd146^{\text{SMC-KO}}$ mice. **d** Characterization of primary murine PASMC isolated from $Cd146^{\text{WT}}$ and $Cd146^{\text{SMC-KO}}$ mice by WB using specific antibodies as indicated. β-actin served as the loading control. *** $P < 0.001$. Two-tailed Student's t-test. All WB represent data from three (**d**) independent experiments. Source data are provided as a Source Data file

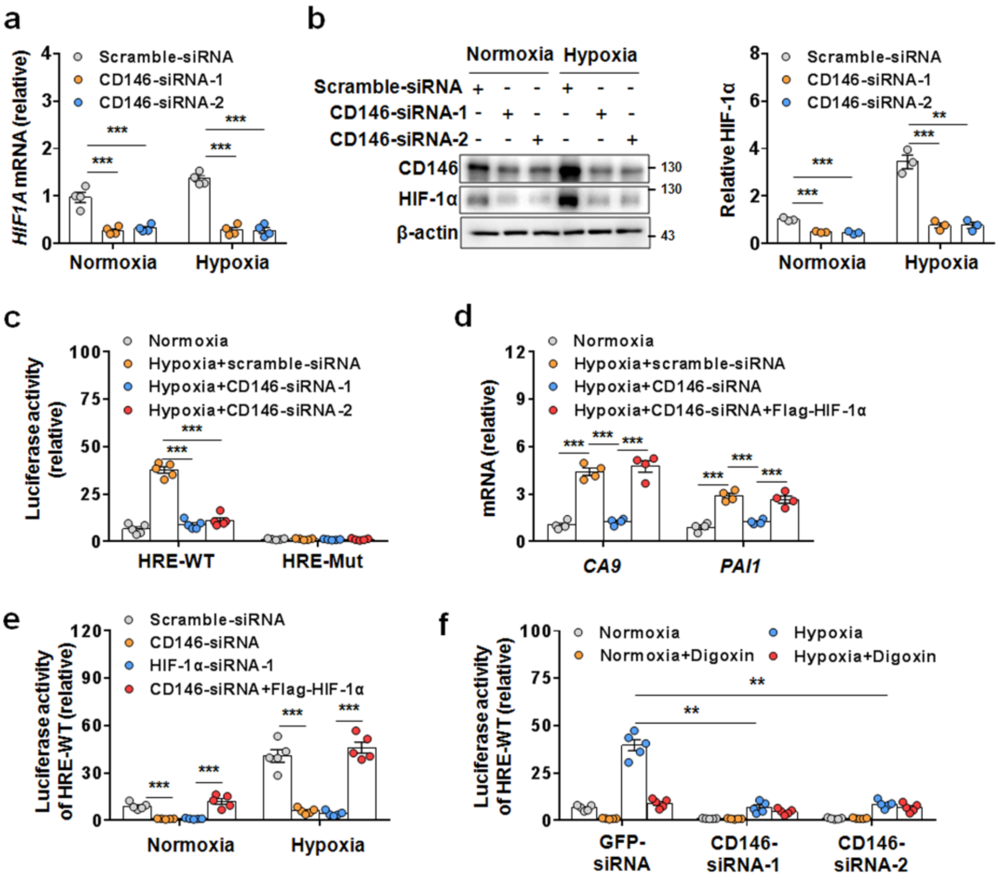

Supplementary Figure 3 CD146 promotes HIF-1α activity under hypoxia. **a**, **b** Human PASMCs were transfected with CD146-siRNAs and exposed to hypoxia for 24 h. HIF-1α expression was measured by real-time RT-PCR (**a**) or WB (**b**). **c** Human PASMCs expressing CD146-siRNAs were cotransfected with indicated reporter constructs before cultured under normoxia or hypoxia for 24 h. Reporter activity was measured and plotted after normalizing with respect to Renilla luciferase activity. **d**, **e** Human PASMCs transfected with CD146-siRNA, HIF-1α-siRNA, or CD146-siRNA and HIF-1α before cultured under normoxia or hypoxia for 24 h. Reporter activity and the mRNA levels of *Ca9* and *Pai1* were detected by real-time RT-PCR (**d**) or luciferase reporter assay (**e**). **f** Human PASMCs transfected with CD146-siRNAs before cultured under normoxia or hypoxia in the presence or absence of digoxin for 24 h. Reporter activity was measured and plotted. β-actin served as the loading control. n = 4 (**a** and **d**) or n = 5 (**c**, **e** and **f**) biological repeats for each group. In all statistical plots, the results are expressed as mean ± s.e.m. ** $P < 0.01$, *** $P < 0.001$. Two-tailed Student's t-test. All WB represent data from three (**b**) independent experiments. Source data are provided as a Source Data file

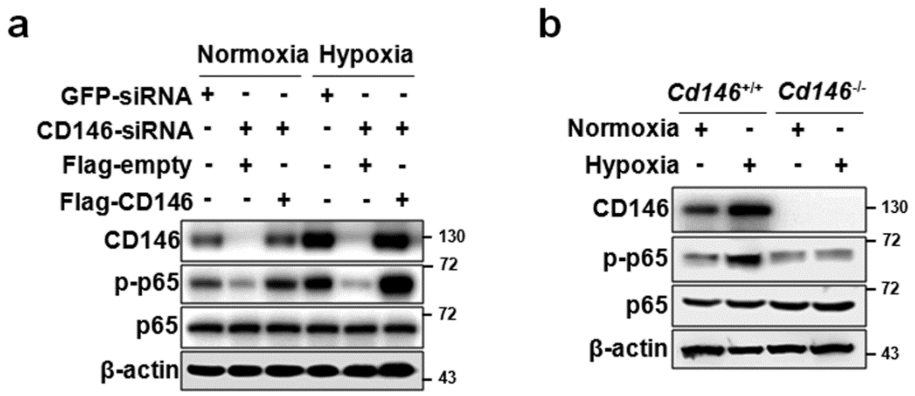

Supplementary Figure 4 CD146 mediates NF-κB activation under hypoxia. **a**, **b** Human (**a**) or mouse (**b**) PASMCs were transfected or treated as indicated. The expression of CD146 and p-p65 were detected by WB. β-actin served as the loading control. All WB represent data are from three (**a**, **b**) independent experiments. Source data are provided as a Source Data file

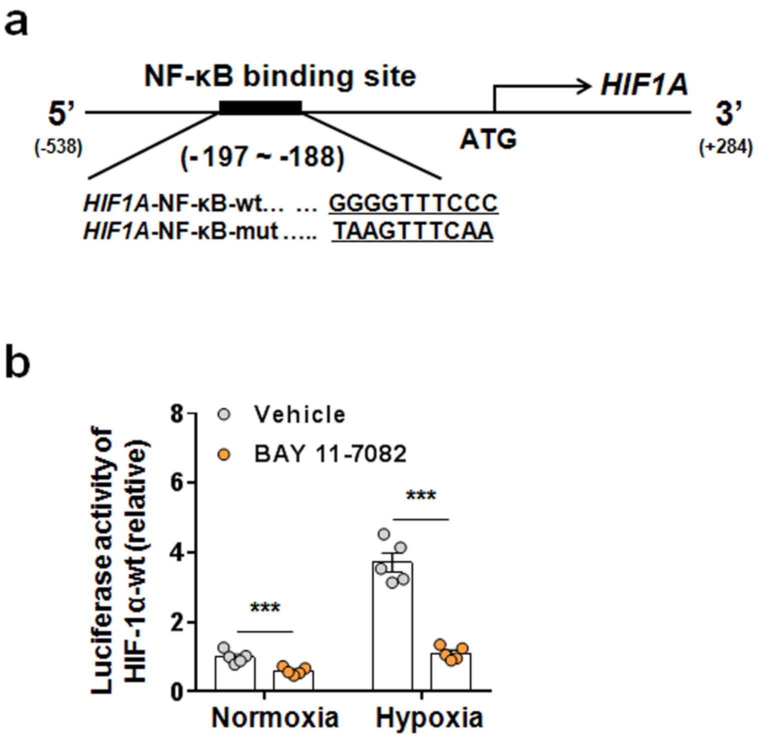

Supplementary Figure 5 Hypoxia activates *HIF1A* transcription via a NF-κB site located at -197/-188 base pairs within the *HIF1A* promoter. **a** Schematic overview of a luciferase construct containing *HIF1A* promoter (-538 to +284) or a mutated NF-κB binding site (-197 to -188) is shown. **b** *HIF1A* promoter-luciferase reporter activity in human PASMCs under normoxia or hypoxia in the presence or absence of the NF-κB inhibitor BAY11-7082, presented relative to luciferase activity in normoxic cells, set as 1. n = 5 (**b**) biological replicates for each group. In all statistical plots, results are expressed as mean ± s.e.m. *** $P < 0.001$. Two-tailed Student's t-test. Source data are provided as a Source Data file

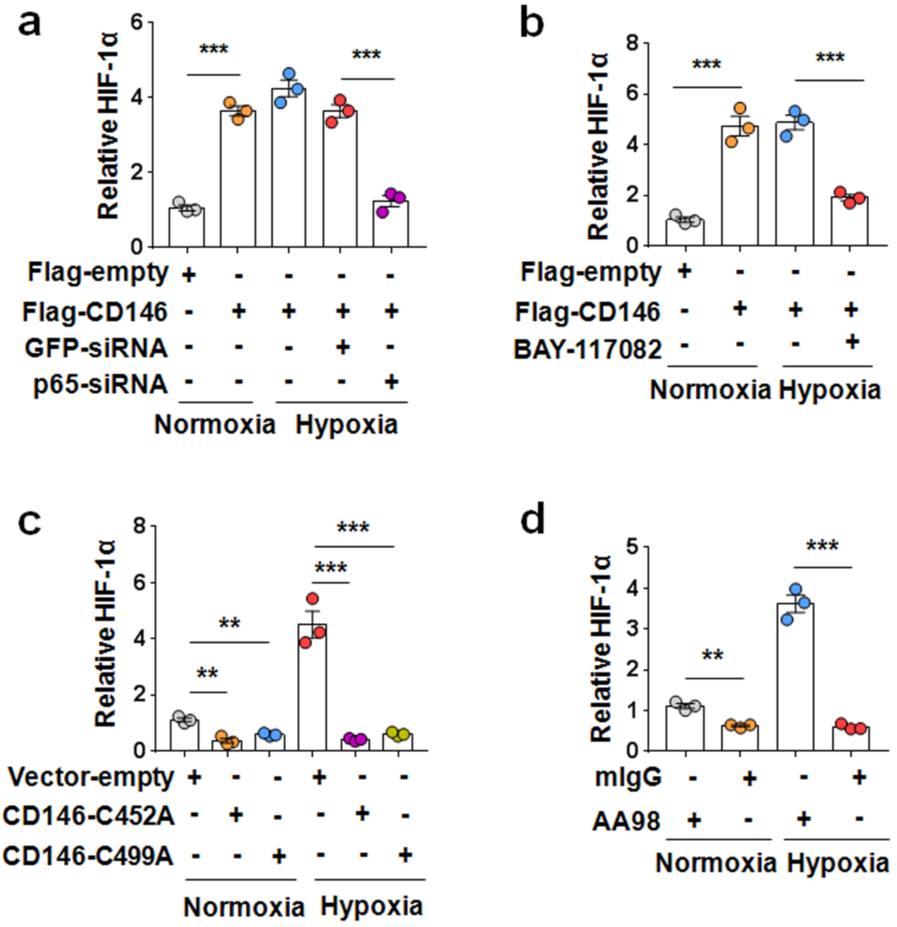

Supplementary Figure 6 Relative expression values obtained by densitometry of HIF-1α protein normalized to β-actin. n = 3 independent experiments. In all statistical plots, the results are expressed as mean ± s.e.m. ** $P < 0.01$, *** $P < 0.001$; by two-tailed Student's t-test. Source data are provided as a Source Data file

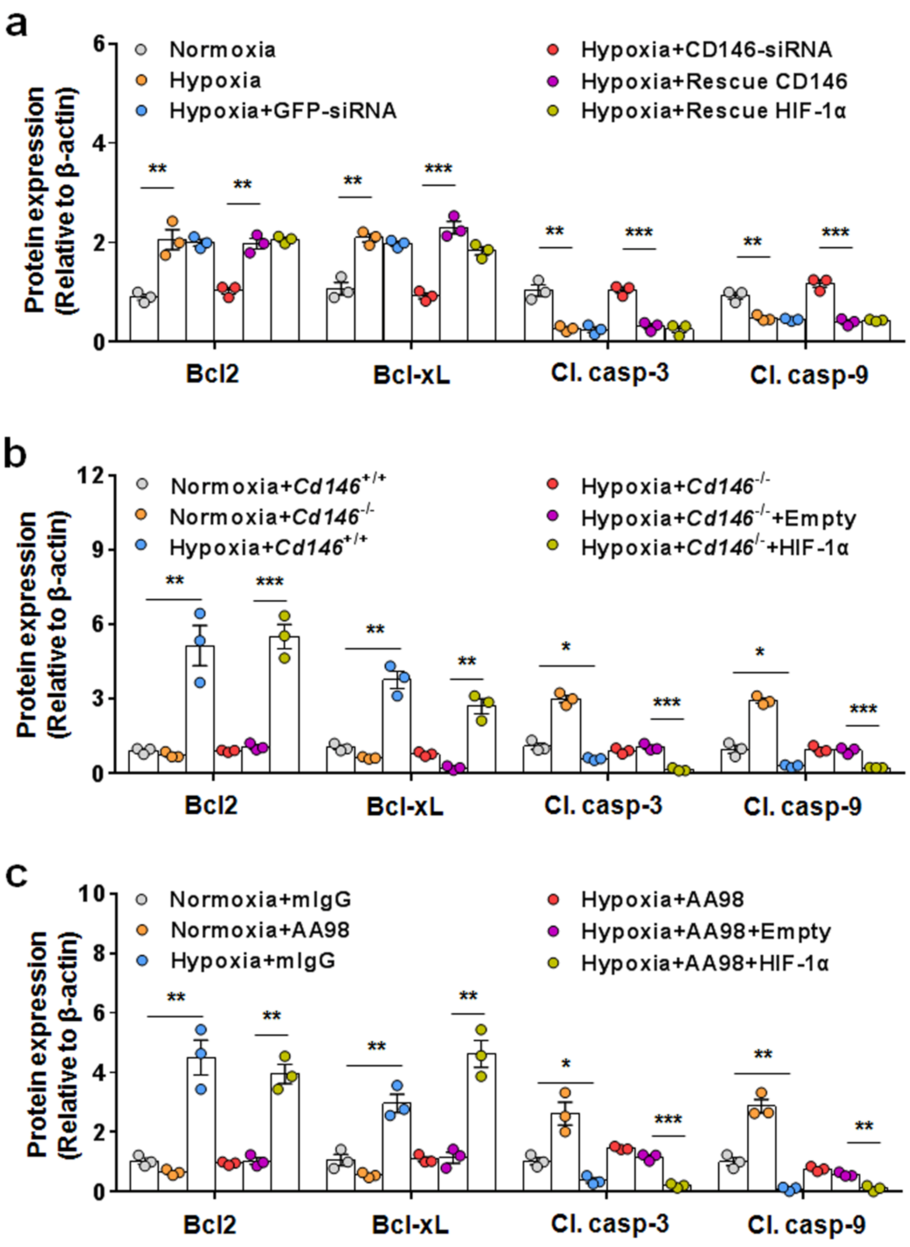

Supplementary Figure 7 Relative expression values obtained by densitometry of indicated proteins normalized to β-actin. n = 3 independent experiments. In all statistical plots, the results are expressed as mean ± s.e.m. ** $P < 0.01$, *** $P < 0.001$; by two-tailed Student's t-test. Source data are provided as a Source Data file

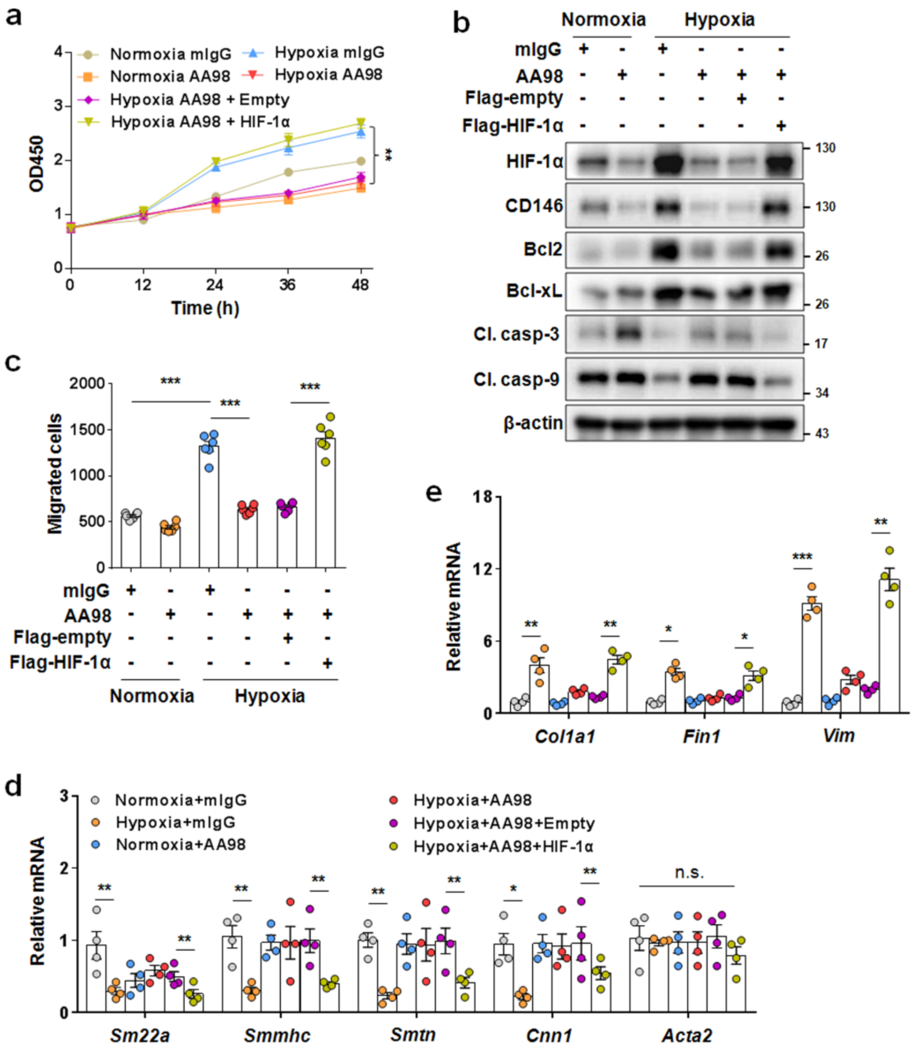

Supplementary Figure 8 Targeting CD146-HIF-1α axis inhibits the synthetic phenotype of cultured mouse PASMCs. **a**, **b** Mouse PASMCs were cultured under normoxic or hypoxic conditions in the presence of anti-CD146 AA98 or control mIgG (50 μg/ml). The cell proliferative ability and the expression of HIF-1α and CD146 were determined by CCK-8 assay (**a**) or WB (**b**). n = 6 (**a**) biological replicates for each group. **c** Mouse PASMCs were treated as in (**a**). Cell migration was measured in a Transwell Boyden chamber. The number of migrated cells was counted. n = 6 biological replicates for each group. **d**, **e** Mouse PASMCs were treated as in (**a**). The mRNA levels of contractile (**d**) and synthetic markers (**e**) were detected by real-time RT-PCR. n = 4 biological replicates for each group. In all statistical plots, the results are expressed as mean ± s.e.m. * $P < 0.05$, ** $P < 0.01$, *** $P < 0.001$. n.s., not significant; by two-tailed Student's t-test. All WB represent data from three (**b**) independent experiments. Source data are provided as a Source Data file

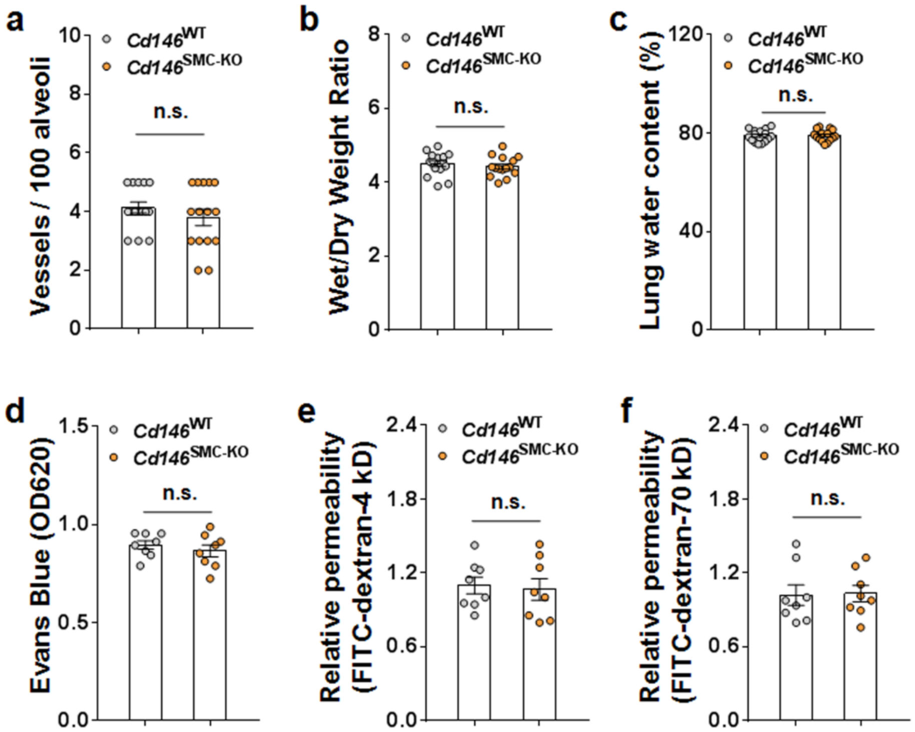

Supplementary Figure 9 Neither vascular number nor integrity was altered in the lung from *Cd146*$^{SMC-KO}$ mice. **a** Quantification of the ratio of vessel/alveoli in the lung of *Cd146*^{WT} and *Cd146*$^{SMC-KO}$ mice (n = 5 mice per group, 3 lung sections per mouse). **b**, **c** Wet/Dry Weight Ratio (**b**) and water content (**c**) of the lung from 8-10 week old *Cd146*^{WT} and *Cd146*$^{SMC-KO}$ mice (n = 15 mice per group). **d-f** *Cd146*^{WT} and *Cd146*$^{SMC-KO}$ mice (8-10 week) were given an i.p. injection or i.v. injection of Evans blue dye or FITC-dextran-4 kD and FITC-dextran-70 kD dye respectively, and the absorption of the dyes extracted from the lung was measured by a microplate spectrophotometer (n = 8 mice per group). In all statistical plots, the results are expressed as mean ± s.e.m. n.s., not significant. Two-tailed Student's t-test. Source data are provided as a Source Data file

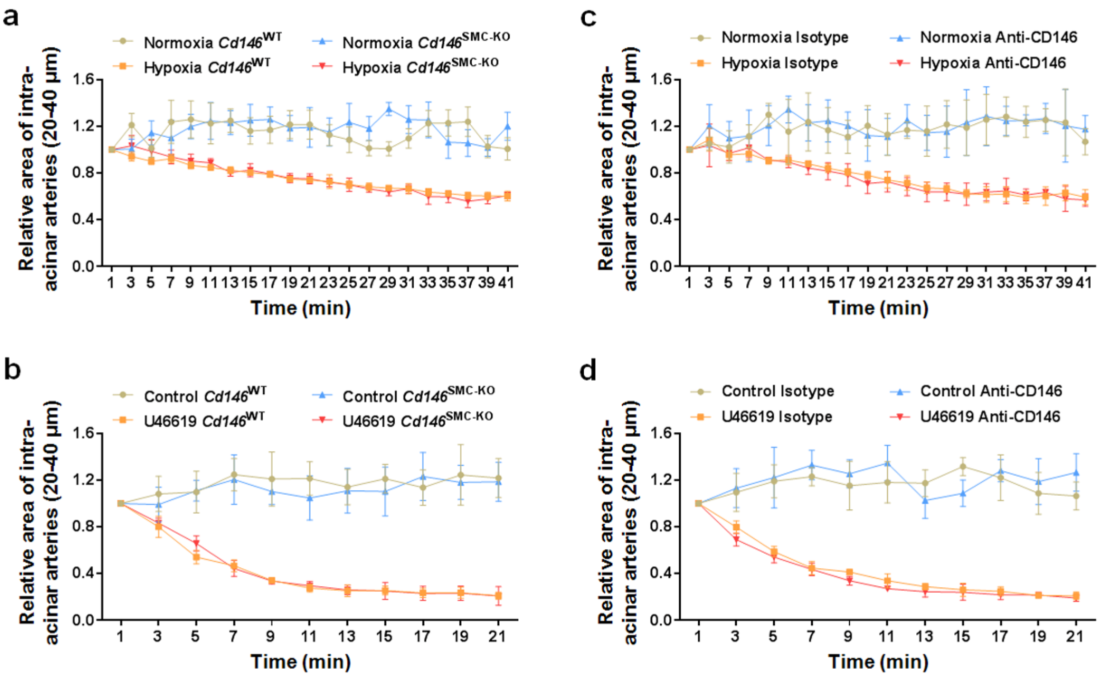

Supplementary Figure 10 Targeting CD146-HIF-1α axis in SMC failed to affect pulmonary vasoconstriction. The cross-sectioned pre-acinar arteries isolated from *Cd146*^{WT} and *Cd146*$^{SMC\text{-}KO}$ mice (**a**, **b**) or from CD146^{WT} mice in the presence of anti-CD146 or mIgG (50 μg/ml) (**c**, **d**) were treated with hypoxia (1% O_2) (**a**, **c**) or U46619 (0.1 μM) (**b**, **d**). Vasoreactivity is recorded as changes in the luminal area, which are plotted against time. The luminal area at the beginning of the experiment is defined as 100% and vasoconstriction induced by hypoxia or U46619 are given as relative values. At least 5 intra-acinar PAs were analyzed. Source data are provided as a Source Data file

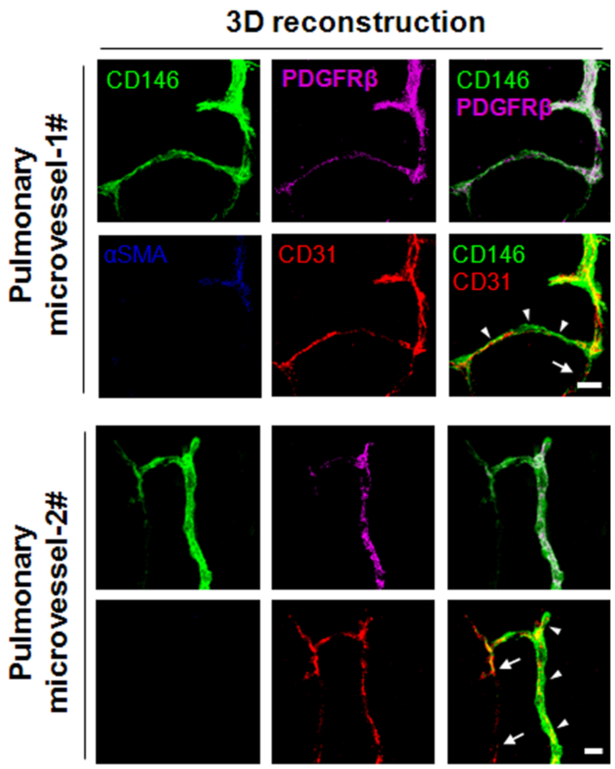

Supplementary Figure 11 Expression of CD146 in pulmonary microvasculature. Lung sections (20 μm thickness) were stained for CD146 (green), CD31 (EC marker, red), perivasulcar markers αSMA (blue) and PDGFRβ (purple). Shown are three-dimensional reconstructions of confocal image z-stacks of pulmonary microvasculature. The expression of CD146 in pulmonary microvasculature was mainly in pericytes but not in pulmonary microvascular endothelial cells (PMVECs) covered with pericytes (arrowheads), whereas PMVECs without pericyte coverage still expressed CD146 (arrows). Scale bars represent 10 μm. At least 10 pulmonary microvessels were analyzed. Two representative pulmonary microvessels were shown.

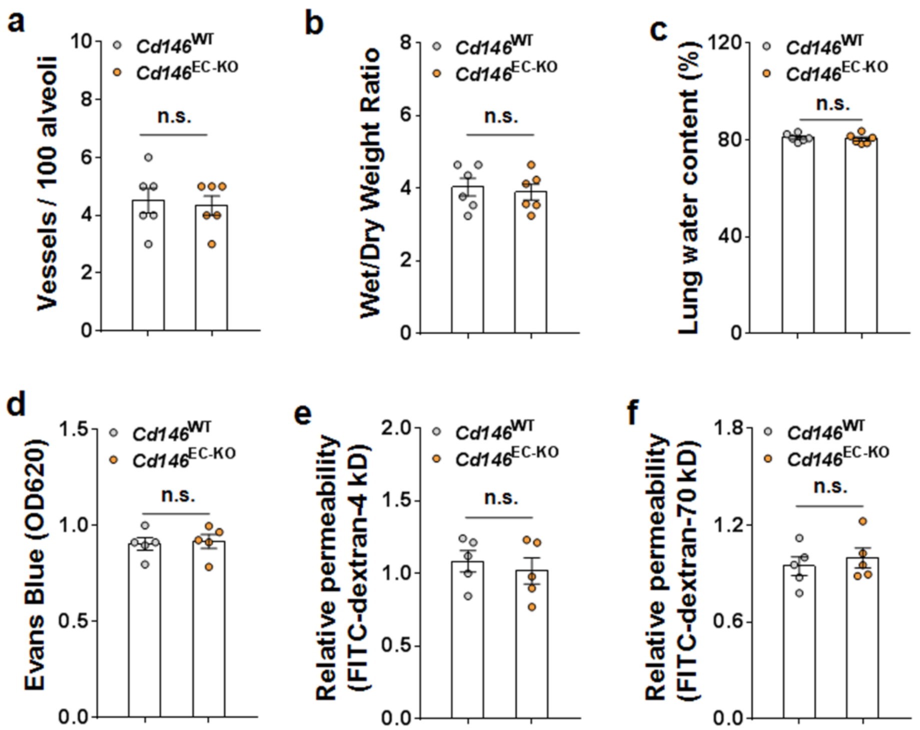

Supplementary Figure 12 Neither vascular number nor integrity was altered in the lung from *Cd146*$^{EC\text{-}KO}$ mice. **a** Quantification of the ratio of vessel/ 100 alveoli in the lung of *Cd146*^{WT} and *Cd146*$^{EC\text{-}KO}$ mice (n = 6 mice per group). **b**, **c** Wet/Dry Weight Ratio (**b**) and water content (**c**) of the lung from 8-10 week old *Cd146*^{WT} and *Cd146*$^{EC\text{-}KO}$ mice (n = 6 mice per group). **d-f** *Cd146*^{WT} and *Cd146*$^{EC\text{-}KO}$ mice (8-10 week) were given an i.p. injection or i.v. injection of Evans blue dye or FITC-dextran-4 kD and FITC-dextran-70 kD dye respectively, and the absorption of the dyes extracted from the lung was measured by a microplate spectrophotometer (n = 5 mice per group). In all statistical plots, the results are expressed as mean ± s.e.m. n.s., not significant. Two-tailed Student's t-test.

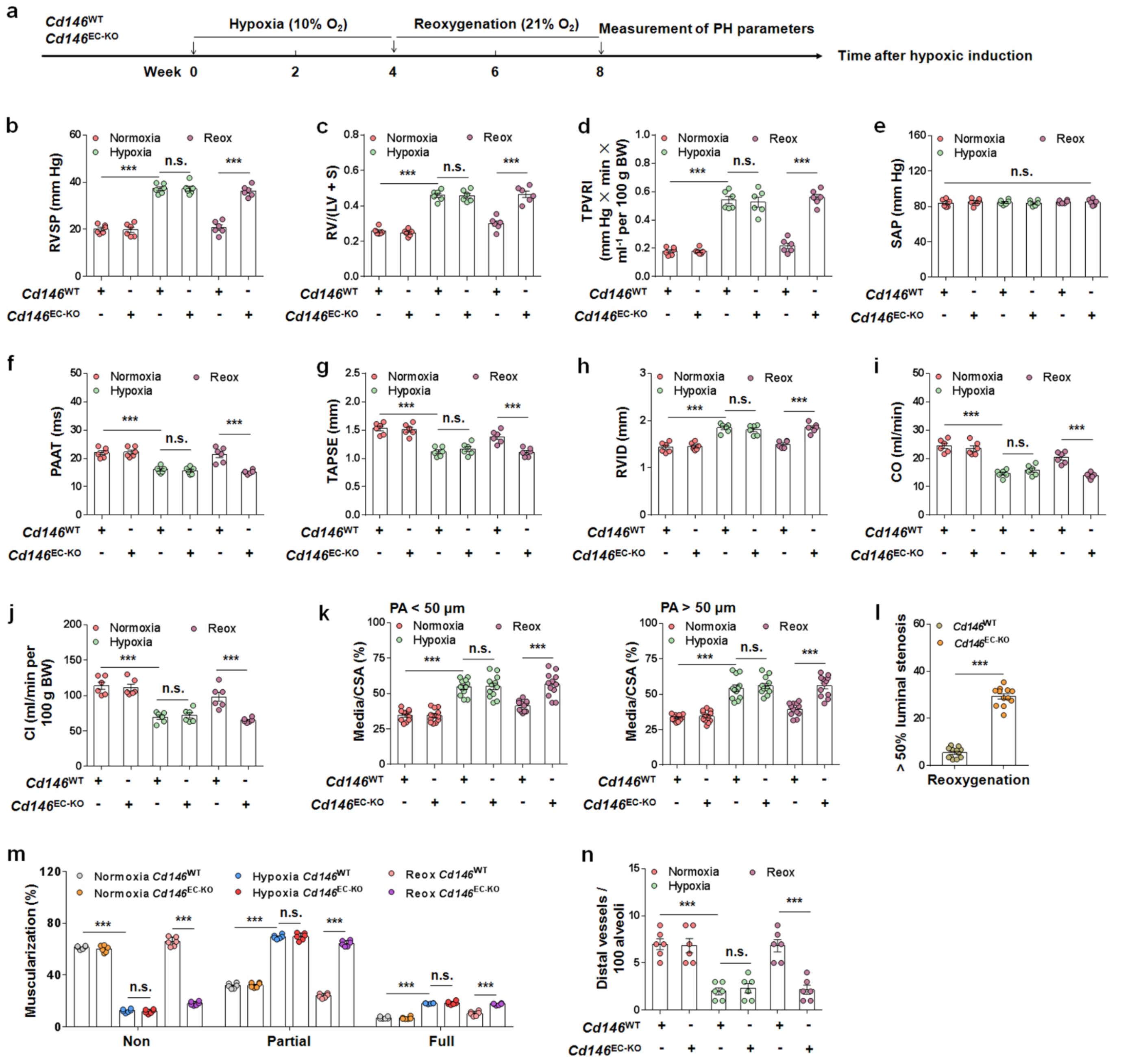

Supplementary Figure 13 *Cd146*$^{EC-KO}$ mice show unresolved PH in hypoxia-reoxygenation. **a** Schematic of hypoxia-induced PH. **b-e** RVSP, RV/(LV + S), TPVRI and SAP in *Cd146*$^{EC-KO}$ and *Cd146*^{WT} mice in normoxia, hypoxia and recovery. **f-j** Quantification of echocardiographic (PAAT, TAPSE, RVID, CO and CI) measurements. **k**, **l** Quantification of vascular medial thickness (**k**) and PAs with > 50% luminal stenosis (**l**) (n = 4 mice per group, 3 PAs per mouse). **m** Proportion of non-, partially-, or fully- muscularized pulmonary arterioles (20-50 μm in diameter). **n** Quantification of the number of distal vessels per 100 alveoli. n = 6 mice per group (**b-j**, **m**, **n**). In all statistical plots, the results are expressed as mean ± s.e.m. ** $P < 0.01$, *** $P < 0.001$. n.s., not significant; by one-way ANOVA with Bonferroni *post hoc* analysis (**b-j**) or two-tailed Student's t-test (**k-n**).

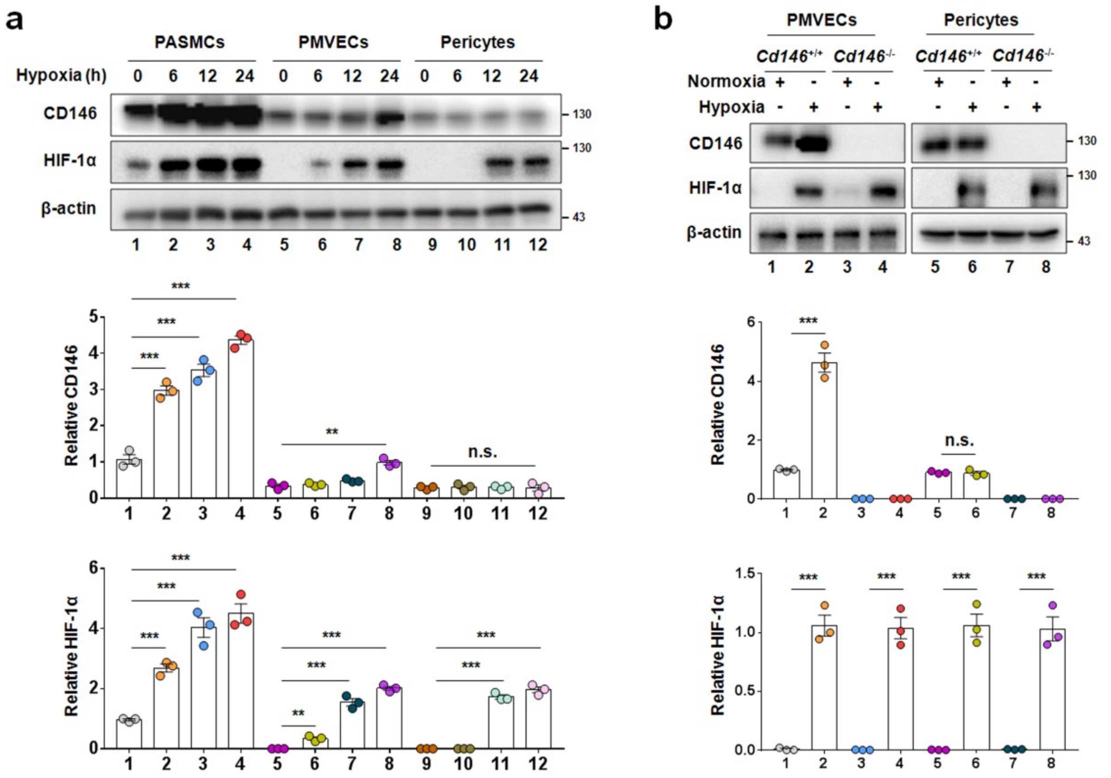

Supplementary Figure 14 Distinct responsiveness and regulation of CD146 and HIF-1α in different types of pulmonary vascular cells. **a** A time-course of the effect of hypoxia on the generation of HIF-1α and CD146 in primary PASMCs, PMVECs and pericytes. The three types of cells isolated from *Cd146*^{WT} mice were treated under normoxia or hypoxia for indicated time points and the expression of CD146 and HIF-1α was measured by WB. Below, quantification of CD146 and HIF-1α expression. **b** The cross-regulation between CD146 and HIF-1α might not be conserved in PMVECs and pericytes. PMVECs and pulmonary pericytes isolated from WT or *Cd146* knockout mice were treated under normoxia or hypoxia for 24h and the expression of CD146 and HIF-1α was measured by WB. Below, quantification of CD146 and HIF-1α expression. In all statistical plots, the results are expressed as mean ± s.e.m. ** P < 0.01, *** P < 0.001. n.s., not significant; by two-tailed Student's t-test. All WB represent data from three independent repeats.

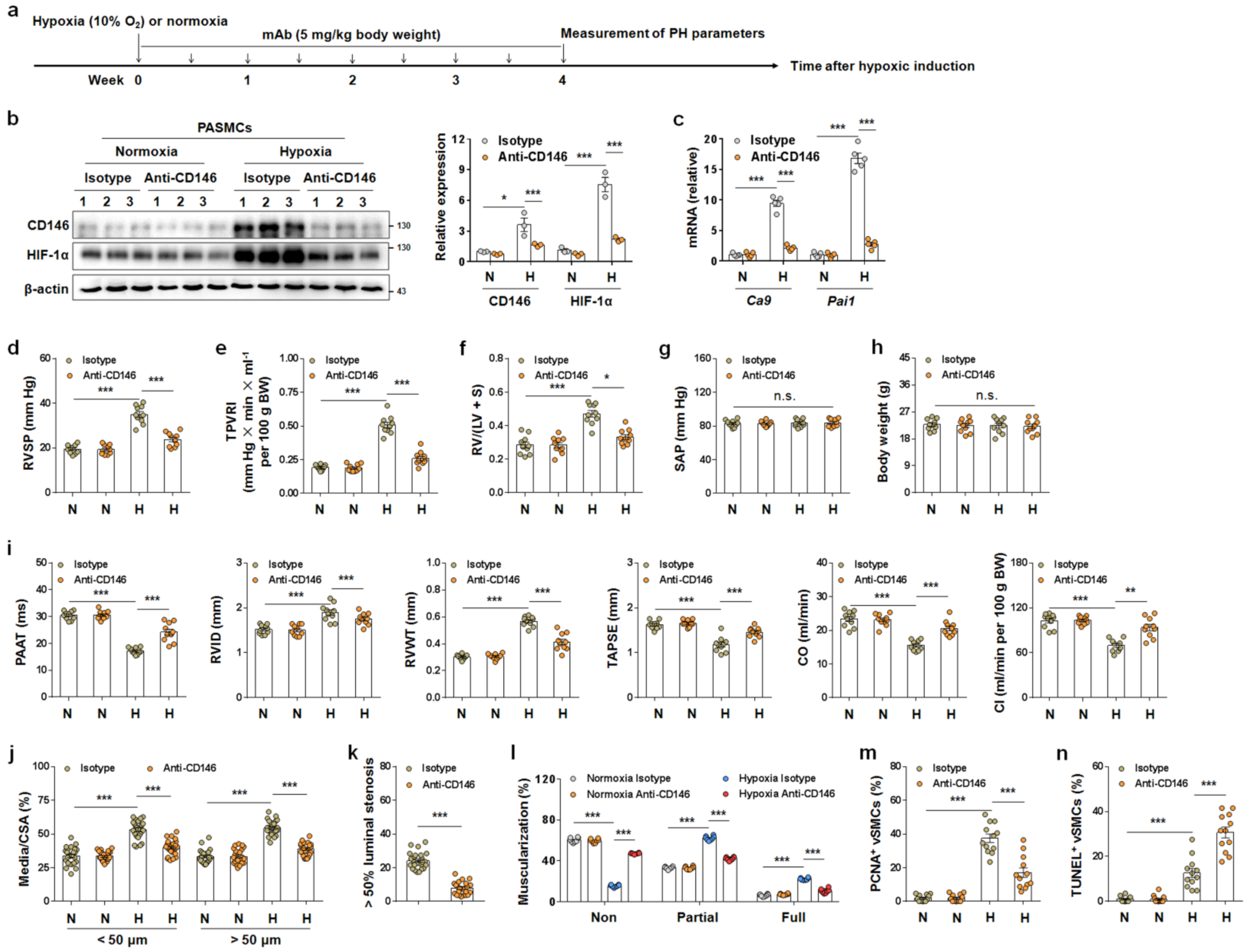

Supplementary Figure 15 Preventive targeting of CD146-HIF-1α axis attenuates hypoxic PH in mice. **a** Schematic of preventive treatment. **b** Left, WB of CD146 and HIF-1α in PASMCs. Right, relative expression of CD146 and HIF-1α (n = 3 biological replicates). **c** The mRNA levels of *Ca9* and *Pai1* in PASMCs were detected by real-time RT-PCR (n = 5 biological replicates). **d-h** RVSP (**d**), TPVRI (**e**), RV/(LV + S) (**f**), SAP (**g**) and body weight (**h**) in anti-CD146- or mIgG-treated mice (n = 10 mice per group). **i** Echocardiography measurements of PAAT, RVID, RVWT, TAPSE, CO and CI (n = 10 mice per group). **j** Quantification of vascular medial thickness (n = 5 mice per group, 5 PAs per mouse). **k** Quantification of PAs with > 50% luminal stenosis (n = 5 mice per group, 5 PAs per mouse). **l** Proportion of non-, partially-, or fully- muscularized pulmonary arterioles (20-50 μm in diameter, n = 8 mice per group). **m** Quantification of the number of $\alpha SMA^+/PCNA^+$ cells (n = 4 mice per group, 3 PAs per mouse). **n** Quantification of the number of $TUNEL^+$ cells (n = 4 mice per group, 3 PAs per mouse). In all statistical plots, the results are expressed as mean ± s.e.m. * $P < 0.05$, ** $P < 0.01$, *** $P < 0.001$. n.s., not significant; one-way ANOVA with Bonferroni *post hoc* analysis (**d-i**) or two-tailed Student's t-test (**b**, **c**, **j-m**). All WB represent data from three (**b**) independent experiments. Source data are provided as a Source Data file

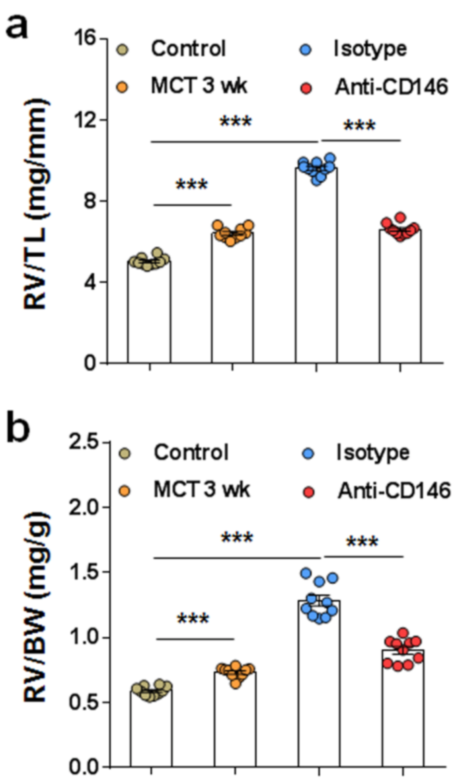

Supplementary Figure 16 Therapeutic targeting of CD146-HIF-1α axis attenuates right heart hypertrophy in MCT-induced PH in rats. RV/TL (**a**) and RV/BW (**b**) in anti-CD146- or isotype-treated rats after 5 weeks of MCT (n = 10 rats per group). In all statistical plots, the results are expressed as mean ± s.e.m. *** $P < 0.001$; one-way ANOVA with Bonferroni *post hoc* analysis (**a**, **b**). TL, tibia length; BW, body weight. Source data are provided as a Source Data file

Supplementary Table 1 Primers designed for real-time PCR analysis of mouse samples

Target gene	sequence (5'-3')
Actb	Forward：GGTGGGAATGGGTCAGAAGG<br>Reverse：GTTGGCCTTAGGGTTCAGGG
Cd146	Forward：CGGGTGTGCCAGGAGAG<br>Reverse：GGCGGTGCTCATATTCACCA
Ca9	Forward：CACCGTTTCCCTGCTGAGAT<br>Reverse：CAGGAATTCACACTTCCTCCGA
Pai1	Forward：TGTGGGGTCACATGGTCTTG<br>Reverse：TACTGCTCGAATCCCTTGCC
Hif1a	Forward：TCCTCCGGATCAGTTCGAGT<br>Reverse：GTGCCACCAGTACATTGGGA
Acta2	Forward：TCCCTGGAGAAGAGCTACGAA<br>Reverse：TACCCCCTGACAGGACGTTG
Cnn1	Forward：CAGATGCATGCCTGGAAACG<br>Reverse：TCTAAGCCATGCTCCTAAACAACT
Smtn	Forward：CCCCGTCTGTCAAAACACCT<br>Reverse：CTGACATCCTCGGGTGACTC
Sm22a	Forward：ATGAGCCGAGAAGTGCAGTC<br>Reverse：ACCTTCACTGGCTTCGATCC
Smmhc	Forward：CGAGGCTGAGAGGGAGCTTG<br>Reverse：AGCCTGTCTCTTGTAGGCTTTC
Col1a1	Forward：TTCCGCTGGGTGTCTGTTAC<br>Reverse：ACCCTCCCTCATTCCTGGTT
Fn1	Forward：GTGCAACTGGAAACGGGAAC<br>Reverse：GCGGGCAAATGCATCTGTAG
Vim	Forward：AGGCCGAGGAATGGTACAAG<br>Reverse：AGTTAGCAGCTTCAAGGGCA

Supplementary Table 2 Primers designed for real-time PCR analysis of human samples

Target gene	sequence (5'-3')
CA9	Forward：CTCGGAGCACACTGTGGAAG<br>Reverse：TTCCAAGCGAGACAGCAACT
PAI1	Forward：CTTGTGGCCGCCTGAGAC<br>Reverse：CCCCTTGCATTTCTGCTCCT
HIF1A	Forward：GTCTGAGGGGACAGGAGGAT<br>Reverse：AAAGGCAAGTCCAGAGGTGG
ACTB	Forward：GGCTCTTTTCCAGCCTTCCT<br>Reverse：GAGCCAGAGCAGTGATCTCC
CD146	Forward：AACACAGTGGGCGCTATGAA<br>Reverse：AACTCGAGGTCCTGGCTACT
COL1A1	Forward：AGACATGGGTTTGGTGACCTG<br>Reverse：GGAACTGCCCAACTCCACTA
FN1	Forward：ACAAGCATGTCTCTCTGCCA<br>Reverse：GCAATGTGCAGCCCTCATTT
VIM	Forward：GTGGACCAGCTAACCAACGA<br>Reverse：AAGGTCAAGACGTGCCAGAG
ACTA2	Forward：CAACCGGGAGAAAATGACTC<br>Reverse：GCGTCCAGAGGCATAGAGAG
CNN1	Forward：GGAGCTGAGAGAGTGGATCG<br>Reverse：CCTGGCTGCAGCTTATTGAT
SMTN	Forward：GGGATCTCACCAGAAAGGAA<br>Reverse：CAGATCTGCTGTGACCTCCA
SM22A	Forward：ATCATAGTGCAGTGTGGCCC<br>Reverse：CACCTGCTCCATCTGCTTGA
SMMHC	Forward：GCATCGGCAGACATCGAAAC<br>Reverse：CGATACTGGCTACCGTGACC

Supplementary Table 3 siRNA sequences designed for this study

Target gene	sequence (5'-3')
HIF1A-1#	Forward：CUGAUGACCAGCAACUUGA
	Reverse：UCAAGUUGCUGGUCAUCAG
HIF1A-2#	Forward：CUGGACACAGUGUGUUUGA
	Reverse：UCAAACACACUGUGUCCAG
CD146-1#	Forward：CCAGCUCCGCGUCUACAAA
	Reverse：UUUGUAGACGCGGAGCUGG
CD146-2#	Forward：GAUGGCAUUCAAGGAGAGGAA
	Reverse：UUCCUCUCCUUGAAUGCCAUC
p65	Forward：AAGGUGCAGAAAGAGGACA
	Reverse：UGUCCUCUUUCUGCACCUU
HIF2A	Forward：AGGUGGAGCUAACAGGACAUA
	Reverse：UAUGUCCUGUUAGCUCCACCU
TP53	Forward：GACUCCAGUGGUAAUCUAC
	Reverse：GUAGAUUACCACUGGAGUC
Control	Forward：CUUCAGCCUCAGCUUGCCG
	Reverse：CGGCAAGCUGACCCUGAAG
